# Temporal Order Judgements of Dynamic Gaze Stimuli Reveal a Postdictive Prioritisation of Averted Over Direct Shifts

**DOI:** 10.1177/2041669517720808

**Published:** 2017-07-17

**Authors:** Nicola Binetti, Charlotte Harrison, Isabelle Mareschal, Alan Johnston

**Affiliations:** UCL Interaction Centre, University College London, UK; Department of Experimental Psychology, University College London, UK; Department of Experimental Psychology, University College London, UK; School of Biological and Chemical Sciences, Queen Mary University of London, UK; Department of Experimental Psychology, University College London, UK; School of Psychology, University of Nottingham, UK

**Keywords:** temporal order judgements, prior entry, stimulus onset asynchrony, gaze shifts, gaze cueing, social communication

## Abstract

We studied temporal order judgements (TOJs) of gaze shift behaviours and evaluated the impact of gaze direction (direct and averted gaze) and face context information (both eyes set within a single face or each eye within two adjacent hemifaces) on TOJ performance measures. Avatar faces initially gazed leftwards or rightwards (Starting Gaze Direction). This was followed by sequential and independent left and right eye gaze shifts with various amounts of stimulus onset asynchrony. Gaze shifts could be either *Matching* (both eyes end up pointing direct or averted) or *Mismatching* (one eye ends up pointing direct, the other averted). Matching shifts revealed an attentional cueing mechanism, where TOJs were biased in favour of the eye lying in the hemispace cued by the avatar’s Starting Gaze Direction. For example, the left eye was more likely to be judged as shifting first when the avatar initially gazed toward the left side of the screen. Mismatching shifts showed biased TOJs in favour of the eye performing the averted shift, but only in the context of two separate hemifaces that does not violate expectations of directional gaze shift congruency. This suggests a postdictive inferential strategy that prioritises eye movements based on the type of gaze shift, independently of where attention is initially allocated. Averted shifts are prioritised over direct, as these might signal the presence of behaviourally relevant information in the environment.

## Introduction

Gaze behaviours guide social interactions and deliver nonverbal information about goals and mental states (Baron-Cohen, 1997). People invest more time looking at the eyes compared with other facial attributes (Janik, Wellens, Goldberg, & Dell'Osso, 1978; Morton & Johnson, 1991), a preference that emerges at early developmental stages (Driver et al., 1999; Klin, Lin, Gorrindo, Ramsay, & Jones, 2009). Direct (or forward—when someone looks straight at us) and averted (when someone is looking away) gaze behaviours deliver information pertinent to different aspects of social communication. Direct gaze is a precursor to most interactions and can be an expression of interest or hostility (Kleinke, 1986). On the other hand, other’s averted gaze can signal the presence of potentially rewarding or threatening stimuli in the environment, providing a basis for joint attention (Frischen, Bayliss, & Tipper, 2007; Sweeny & Whitney, 2014).

Being able to adequately classify direct and averted gaze signals requires mechanisms for processing directional gaze information (Ewbank, Jennings, & Calder, 2009; Mareschal, Calder, Dadds, & Clifford, 2013). Imaging and electrophysiological data provide evidence of dedicated neural systems for gaze direction processing in the primate and human brain. Human functional imaging studies have documented posterior superior temporal sulcus (STS) activation in gaze processing (Hoffman & Haxby, 2000; Pelphrey, Morris, & McCarthy, 2005) and distinct neural populations in the anterior STS and inferior temporal lobule tuned to different gaze shift directions (Calder et al., 2007). Electroencephalographic evidence has indicated differences in brain activity when observing direct opposed to averted gaze (Conty, N’Diaye, Tijus, & George, 2007; McCarthy, Puce, Belger, & Allison, 1999). In vivo recordings in the anterior STS of macaque monkeys have identified cell populations which selectively respond to direct and averted gaze stimuli (Perrett & Emery, 1994; Perrett et al., 1985).

Direct and averted signals are however embedded within dynamic gaze behaviours; evaluating directional information is equally important to determining when shifts occur, in what order and how long they last. In previous studies, we have addressed the mechanisms through which people estimate the duration of gaze behaviours. In a 500-participant sample, covering a wide range of ages and nationalities, we measured what duration constitutes a ‘normal’ amount of eye contact and related individual preferences in gaze duration to changes in pupil dilation (a proxy for physiological arousal; Binetti, Harrison, Coutrot, Johnston, & Mareschal, 2016). This revealed a period of roughly 3 seconds as a comfortable amount of eye contact which was largely independent of participant demographic and personality variables. We also found that the rate at which pupil size increases predicts people’s preferred eye contact duration: Participants who preferred longer periods of eye contact exhibited faster rates of pupil increase. In a more recent study, we have shown with pupil response measures that people exploit internal arousal signals to time gaze behaviours in others (Binetti et al., 2017). This was not observed when participants evaluated the duration of equivalent neutral spatial displacements (Gabor shifts), thus providing the first evidence of dedicated timing machinery for estimating the duration of gaze behaviours.

In the present study, we investigate the mechanisms that inform temporal order judgements (TOJ) of dynamic gaze shifts performed by avatar face stimuli. Avatars initially looked toward the left or right side of the screen and after a variable delay performed *Matching* direct or averted gaze shifts (both eyes end up pointing direct or averted) or *Mismatching* shifts (one eye shifts to a direct direction, the other to an averted direction). We introduced an asynchrony between the left and right eye shifts, and asked participants to indicate which eye shifted earlier (TOJ). We assessed how TOJs were modulated by directional cue information prior to the avatar’s gaze shifts (starting gaze direction), by the type of gaze shifts the avatars performed (direct or averted shifts) and by the face context within which these shifts were set (left and right eyes set within a single face or each eye set within one of two adjacent hemifaces). We manipulated starting gaze direction to study the effect of attentional constraints on gaze shift TOJs. Prior research has shown that starting gaze provides a strong form of attentional cueing (Driver et al., 1999; Schneider & Bavelier, 2003): attention is automatically drawn in the direction the eyes are pointing. Since spatial attention biases TOJs (i.e. the object of attention appears to occur first, i.e. ‘prior entry’), we expect on the basis of this that eye shift TOJs will be biased in favour of the eye lying in the hemispace cued by the avatar’s starting gaze direction. For example, participants might have a tendency to report the left eye as shifting earlier when the avatar is initially looking toward the left side of the screen (drawing attention toward the left hemispace). We also manipulated gaze shift direction to evaluate how TOJs are affected by the directional content of left and right eye shifts. Several studies have highlighted asymmetries in the processing of direct and averted stimuli. Direct gaze is known to enhance attention and cognition (Conty et al., 2007; Senju & Hasegawa, 2005). We evaluated four gaze shift combinations: two directionally Matching shifts, where both eyes shift to direct (DD) or both shift to averted (AA); and two directionally Mismatching shifts, where the left eye shifts to direct and the right to averted (DA) or the left eye shifts to averted and the right to direct (AD). When eyes perform Matching shifts, we expect that direct shifts (DD) will lead to improved TOJ performance relative to averted shifts (AA), that is, greater precision and sensitivity to differences in TOJs. When eyes perform Mismatching shifts, where direct and averted both occur within the same trial, we predict that TOJs will be biased toward the eye performing the direct shift, that is, leftward bias in DA trials and rightward bias in AD trials. Finally, we manipulated face context information to examine potential benefits in temporal order processing of features belonging to a single object or to two separate objects. One might expect on the grounds of object-based attention (Duncan, 1984; Kimchi, Yeshurun, & Cohen-Savransky, 2007), and the appreciation that eye movements are coordinated within a face rather than between faces, some facilitation of TOJs in the case of movements of the eyes with the same face as compared with across faces.

## Methods

### Participants

Ten participants were recruited in the study (five Female; age = 33.5 ± 11.5 years, range = 25–61 years). The sample size was based on comparable number of participants tested in previous TOJ studies (Driver et al., 1999; Schneider & Bavelier, 2003; Shore, Spence, & Klein, 2001). All participants had normal or corrected to normal vision. Informed consent was obtained from all participants prior to starting the experiment. The study was approved by the University College London Research ethics committee and was in agreement with the University College London research guidelines and regulations.

### Apparatus

The study was conducted in a dimly lit testing environment. A chinrest restrained head position and stimuli were displayed on a Mitsubishi Diamond Plus 250SB CRT monitor (1280 × 1024 @85 Hz) positioned at a 57-cm viewing distance. Stimulus presentation and response collection were implemented on MATLAB 2013a (Mathworks), with the Psychtoolbox 3 library running on a DELL precision T3500. Avatar stimuli were created with Poser 9 Pro (SmithMicro Software).

### Task and Stimuli

**Figure 1.**
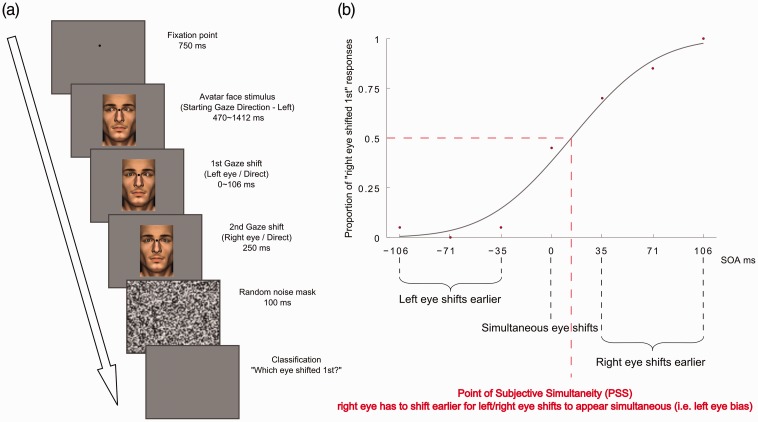
(a) Time course of events in trial. In this specific example, the one face avatar stimulus is selected with a leftward starting gaze direction and both eyes perform a direct gaze shift (the left eye shifts before the right). (b) Psychometric fit of one participant’s responses as a function of SOA. Point of Subjective Simultaneity (PSS), measured in ms: SOA required for the left eye and right eye shifts to appear simultaneous. PSS values > 0 indicate that Temporal Order Judgements (TOJs) are biased in favour of the left eye (the right eye has to shift earlier to generate a percept of simultaneity) while PSS values < 0 indicate that TOJs are biased in favour of the right eye (the left eye has to shift earlier to generate a percept of simultaneity).

At the beginning of each trial, a circular fixation point was displayed on the screen. Following a 750-ms delay, the avatar face stimuli (one face or two adjacent hemifaces) were presented on the screen with left and right eyes pointing either leftwards (toward the left side of the screen, from the participant’s perspective) or rightwards (toward the right side of the screen, from the participant’s perspective; Figure 1(a)). A black fixation point was level with the nasion region (i.e. on the nasion region in the one face condition or in between nasion regions in the two hemiface condition). Avatar stimuli measured approximately 7 cm (Width) × 11 cm (Height), with the eye stimuli set 3.7 cm apart (distance measured from the centre of each eye). The two hemiface stimulus was constructed by swapping the left and right sides of the one face stimulus while ensuring that the distance between eyes remained constant. The initial gaze direction of the avatar stimulus was counterbalanced across blocks. After a variable 470- to 1412-ms delay (sampled from a uniform distribution), the left and right eyes performed gaze shifts (direct or averted) in the direction opposite to the starting gaze direction. The temporal order between left and right eye shifts was varied across seven possible levels of stimulus onset asynchrony (SOA; −106, −71, −35, 0, 35, 71 and 106 ms), randomly selected within each trial. Negative SOA values correspond to the left eye shifting first (Figure 1(b)). A brief 100-ms full-screen random noise mask, smoothed with a Gaussian filter, was presented 250 ms after the presentation of the second gaze shift. The mask was used to minimise the persistence of afterimages. Participants were then required to indicate with an unspeeded button press on the keyboard which eye, left or right (‘a’ or ‘l’ keys, respectively), shifted earlier (TOJ). The next trial started immediately after the participant’s button press. The position of the stimulus on the screen varied across trials, requiring participants to saccade on each occasion to the repositioned fixation cross. The stimuli were randomly displaced within a 50 × 50 pixel area relative to the centre of the screen. This was included to ‘refresh’ stimulus presentation across successive trials, avoiding the emergence of habituation effects that might hinder the processing of the avatar’s face information. We used a single male avatar face throughout, presented on a mid-grey background.

**Figure 2.**
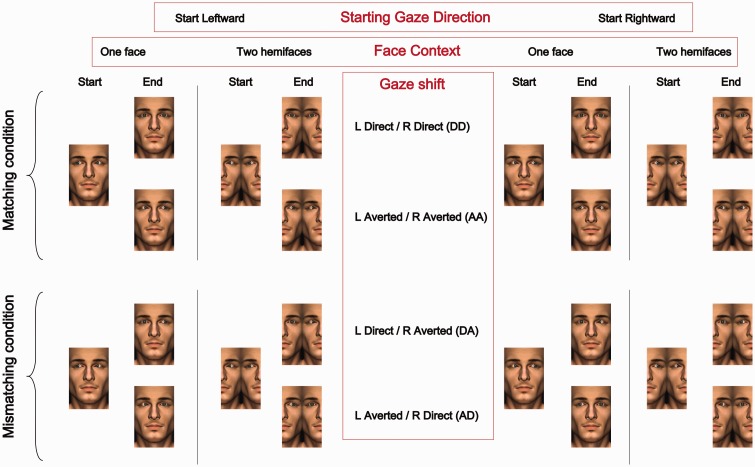
Experimental conditions based on the combinations of Starting Gaze Direction (Leftward or Rightward), Face context (One face or Two hemifaces) and Gaze shifts (Matching: both eyes direct or both eyes averted; Mismatching: one eye performs a direct shift, the other an averted shift). ‘Start’ indicates the avatar’s left/right eye gaze direction prior to the gaze shifts and ‘End’ indicates the avatar’s left/right eye gaze direction after the gaze shifts have taken place. The SOA values determine in what order and with what relative latency these gaze shifts occur (not depicted in the figure).

On each trial, the eyes either performed Matching gaze shifts (both eyes final pointing direction is direct or averted) or Mismatching shifts (the left eye’s final pointing direction is direct and the right eye’s final direction is averted, *or*, the left eye ends up pointing away and the right eye points direct). Participants performed 20 repetitions for each combination of avatar *Starting Gaze Direction* (avatars start gazing leftwards or rightwards), *Face Contexts* (one face/two hemifaces), *Gaze Shift* combination (Matching: Left Direct & Right Direct, Left Averted & Right Averted; Mismatching: Left Direct & Right Averted, Left Averted & Right Direct) and level of SOA (−106, −71, −35, 0, 35, 71 and 106 ms; Figure 2).

### Analysis

We fit participants’ proportion of ‘right eye shifted first’ responses as a function of SOA with a cumulative Gaussian. The 50% point of this function yielded an estimate of the participant’s Point of Subjective Simultaneity (PSS), that is, the amount of asynchrony between left eye and right eye gaze shifts required for these events to be perceived as synchronous (Figure 1(b)). The sign of PSS values reveal biased TOJ in favour of the left or right eye. For example, if the PSS is 50 ms, this means that the right eye shift has to precede the left eye shift by 50 ms in order for the two events to appear synchronous, thus revealing a bias to perceive the left eye as having shifted first. We also calculated the standard deviation (*SD*) of the Gaussian fit as an index of participant sensitivity to variations in SOA. Based on our predictions, we assessed variations in PSS and *SD* separately for Matching and Mismatching conditions. This was aimed at testing differences between DD and AA trials (with congruent directional information), or between DA and AD trials (with conflicting directional information), as a function of starting gaze direction and face context.

#### Matching condition

We submitted the PSS and SD values to a repeated measures analysis of variance (ANOVA), with avatar *Starting Gaze Direction* (avatars start gazing toward left or right side of screen) × *Face Context* (one face / two hemifaces) × *Gaze Shift* (DD: both eyes direct, or AA: both averted) as factors.

#### Mismatching condition

We submitted PSS and SD values to a repeated measures ANOVA, with avatar *Starting Gaze Direction* (avatars start gazing toward left or right side of screen) × *Face Context* (one face / two hemifaces) × *Gaze Shift* (DA: left eye direct / right eye averted, or AD: left eye averted / right eye direct) as factors.

## Results

### Matching Condition

**Figure 3.**
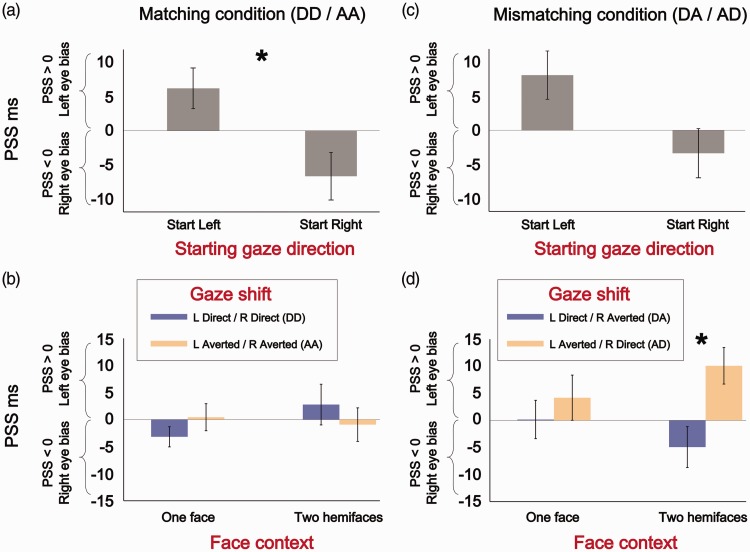
(a) Average PSS values as a function of Starting Gaze Direction with Matching gaze shifts (both eyes direct or both eyes averted shifts). Error bars represent standard error of the mean. TOJs are biased toward the left eye when the avatar initially holds a leftward gaze, while TOJs are biased toward the right eye when the avatar initially holds a rightward gaze. (b) PSS values as a function of Face context and Gaze shift in the Matching condition (nonsignificant interaction). (c) PSS values as a function of Starting Gaze Direction with Matching gaze shifts (one eye performs a direct shift, the other an averted shift). Loss of a significant influence of the avatar’s Starting Gaze direction on TOJs. (d) Significant interaction of Face context and Mismatching Gaze shifts. TOJs are biased in favour of the eye performing an averted gaze shift (the right eye in DA trials; the left eye in AD trials). This is only observed in the two hemiface context.

A 2 × 2 × 2 repeated measures ANOVA run on PSS values only revealed a main effect of *Starting Gaze Direction*, *F*(1, 9) = 6.27, *p* = .03, η_p_^2 ^= .41. PSS signs were modulated by the initial gaze direction of the avatar stimuli. When the avatars initially gazed toward the left side of the screen, TOJs were biased in favour of perceiving the left eye as having shifted first (positive PSS values), while conversely, when the avatars initially gazed toward the right side of the screen, TOJs were biased in favour of the right eye having shifted first (negative PSS values; Figure 3(a)). We observed no significant main effects of *Face Context*, *F*(1, 9) = .001, *p* = .97, η_p_^2 ^= 0, or *Gaze Shift*, *F*(1, 9) = .57, *p* = .47, η_p_^2 ^= .06. No significant interactions were observed: *Starting Gaze Direction* × *Gaze Shift*, *F*(1, 9) = 4.17, *p* = .07, η_p_^2 ^= .32, *Face Context* × *Gaze Shift*, *F*(1, 9) = 2.26, *p* = .167, η_p_^2 ^= .2, *Starting Gaze Direction* × *Face Context*, *F*(1, 9) = .344, *p* = .57, η_p_^2 ^= .04 and *Starting Gaze Direction* × *Gaze Shift* × *Face Context*, *F*(1, 9) = .39, *p* = .55, η_p_^2 ^= .04; Figure 3(b).

A 2 × 2 × 2 repeated measures ANOVA run on *SD* values showed a main effect of *Face Context*, *F*(1, 9) = 5.42, *p* = .04, η_p_^2 ^= .38. Sensitivity to variations in SOA was higher when gaze shifts were embedded within the one face context with respect to the two hemiface context. No significant main effects of *Starting Gaze Direction*, *F*(1, 9) = 2.3, *p* = .16, η_p_^2 ^= .2, or *Gaze Shift*, *F*(1, 9) = 2.2, *p* = .17, η_p_^2 ^= .2, were observed. No significant two-way interactions were observed: *Starting Gaze Direction* × *Gaze Shift*, *F*(1, 9) = .11, *p* = .74, η_p_^2 ^= .01, *Face Context* × *Gaze Shift*, *F*(1, 9) = .1, *p* = .75, η_p_^2 ^= .01, *Starting Gaze Direction* × *Face Context*, *F*(1, 9) = 0, *p* = .99, η_p_^2 ^= 0. A borderline three-way *Starting Gaze Direction* × *Gaze Shift* × *Face Context*, *F*(1, 9) = 5.33, *p* = .047, η_p_^2 ^= .371, revealed a marginally greater difference in *SD* scores between DD trials starting leftwards and DD trials starting rightwards, in the one face condition.

### Mismatching Condition

A 2 × 2 × 2 repeated measures ANOVA run on PSS values in the Mismatching condition showed a similar, but nonsignificant trend of *Starting Gaze Direction* to what was observed in the Matching condition, *F*(1, 9) = 3.89, *p* = .08, η_p_^2 ^= .3 (Figure 3(c)). No significant main effects of *Face Context*, *F*(1, 9) = .03, *p* = .87, η_p_^2 ^= .003, or *Gaze Shift*, *F*(1, 9) = 3.27, *p* = .1, η_p_^2 ^= .27, were observed. We found a significant *Face Context* × *Gaze Shift* interaction, *F*(1, 9) = 6.8, *p* = .03, η_p_^2 ^= .43. Bonferroni corrected post hoc comparisons revealed that PSS values significantly differed between the two *Gaze Shift* conditions (left eye averted / right eye direct vs. left eye direct / right eye averted) only in the context of two separate hemifaces, *t*(18) = 2.96, *p* = .008, critical *p* = .0125, *d* = .86. This significant difference in PSS values revealed that TOJs were biased in favour of the eye performing the averted shift. When the left and right eyes, respectively, performed an averted and direct gaze shift, TOJs were biased in favour of the left eye (positive PSS value), while conversely, when the left and right eyes, respectively, performed a direct and averted shift, TOJs were biased in favour of the right eye (negative PSS values; Figure 3(d)). No significant dissociation in PSS values was observed across *Gaze Shifts* in the one face context, *t*(18) = .74, *p* = .47, *d* = .22. No other significant interactions were observed: *Starting Gaze Direction* × *Gaze Shift*, *F*(1, 9) = 1.8, *p* = .21, η_p_^2 ^= .17, *Starting Gaze Direction* × *Face Context*, *F*(1, 9) = .13, *p* = .72, η_p_^2 ^= .01, and *Starting Gaze Direction* × *Gaze Shift* × *Face Context*, *F*(1, 9) = .01, *p* = .92, η_p_^2 ^= .001.

A 2 × 2 × 2 repeated measures ANOVA run on *SD* values revealed no significant main effects or interactions: *Starting Gaze Direction*, *F*(1, 9) = 3.17, *p* = .11, η_p_^2 ^= .26, *Face Context*, *F*(1, 9) = .68, *p* = .43, η_p_^2 ^= .07, *Gaze Shift*, *F*(1, 9) = .61, *p* = .45, η_p_^2 ^= .06, *Starting Gaze Direction* × *Gaze Shift*, *F*(1, 9) = 1.51, *p* = .25, η_p_^2 ^= .14, *Face Context* × *Gaze Shift*, *F*(1, 9) = .36, *p* = .56, η_p_^2 ^= .04, *Starting Gaze Direction* × *Face Context*, *F*(1, 9) = 1.6, *p* = .24, η_p_^2 ^= .15, and *Starting Gaze Direction* × *Gaze Shift* × *Face Context*, *F*(1, 9) = .51, *p* = .5, η_p_^2 ^= .05.

### Matching Versus Mismatching Condition

We also directly compared Matching and Mismatching PSS values in order to test whether the effect of Starting Gaze Direction was stronger in Matching (DD and AA) opposed to Mismatching (DA and AD) trials. A nonsignificant Starting Gaze Direction versus Match/Mismatch interaction, *F*(1, 9) = .24, *p* = .64, revealed that the influence of Starting Gaze Direction was not significantly stronger in Matching opposed to Mismatching trials.

## Discussion

In this study, we examined how people evaluate the temporal order of asynchronous gaze shifts performed by avatars. We assessed how TOJs were modulated by directional cue information prior to gaze shifts, by the type of gaze shifts the avatars performed (direct or averted), and by the face context within which these shifts were set. When avatars performed Matching gaze shifts (both eyes performed direct or averted shifts), we found that TOJs were biased in favour of the eye lying in the hemispace cued by the avatar’s initial gaze direction, which suggests an attentional cuing phenomenon. For example, if the avatar initially gazed toward the left side of the screen, it was more likely for participants to report the left eye gaze shift as occurring earlier than the right eye shift. We observed a similar, but nonsignificant trend in the Mismatching conditions (one eye performed a direct shift, the other averted)*.* Mismatching shifts also revealed that eye behaviours were prioritised based on the type of gaze shift performed: contrary to our initial prediction, we found that averted shifts appeared to temporally precede direct shift behaviours. This only occurred when gaze shifts were set within the two hemiface context. Most importantly, this was independent of where attention was initially allocated, thus suggesting that a retrospective appraisal of the directional content of eye shifts informs TOJs of gaze behaviour.

The determinants of temporal order perception have been investigated across crossmodal (Frey, 1990; Sternberg & Knoll, 1973), attentional orienting (Shore et al., 2001; Yates & Nicholls, 2009) and saccadic suppression studies (Binda, Cicchini, Burr, & Morrone, 2009; Morrone, Ross, & Burr, 2005). These studies reveal that TOJs are based on information pooled from a variety of different sources (Kresevic, Marinovic, Johnston, & Arnold, 2016; Matthews, Welch, Achtman, Fenton, & FitzGerald, 2016). A first determinant is clearly represented by the times at which sensory signals reach the cortex (Arnold & Wilcock, 2007; Hirsh & Sherrick Jr., 1961; Roufs, 1963). This component is however modulated by attentional gating mechanisms, where TOJs are biased in favour of attended stimuli. This is formalised in Titchener’s (1908) law of prior entry, which states that ‘the object of attention comes to consciousness more quickly than the objects which we are not attending to’ p.251. This implies that an attended stimulus should be presented later in time with respect to an unattended stimulus in order to generate a perception of simultaneity (summarised by PSS values; Spence & Parise, 2010). Several studies have reported asymmetries in perceptual and/or attentional processing between right and left visual hemifields (Battelli, Pascual-Leone, & Cavanagh, 2007; Müri et al., 2002; Śmigasiewicz & Möller, 2011): Stimuli lying in the left hemifield are frequently processed faster (Forster, Corballis, & Corballis, 2000; Woo, Kim, & Lee, 2009), or appear to occur earlier (Matthews & Welch, 2015; Matthews et al., 2016) than stimuli in the right hemifield. We observed, at least in the Mismatching conditions (Figure 3(c) and (d)), a left-eye bias trend (the avatar’s left eye, from the participant’s perspective): mean PSS values (i.e. the mean PSS of Start Left and Start Right trials in Figure 3(c); the mean PSS of DA and AD trials within one face and two hemiface contexts in Figure 3(d)) lie above the point of physical simultaneity (mean PSS > 0 ms). Curiously, this pattern was not observed in the Matching conditions (Figure 3(a) and (b)). Studies have also shown that both exogenous (automatic) and endogenous (voluntary) spatial shifts of attention can determine prior entry effects (Shore et al., 2001; Yates & Nicholls, 2009). Exogenous cues (e.g. a peripheral flash) modulate TOJs by automatically drawing attention and priming sensory processing at cued spatial locations. On the other hand, endogenous cues (e.g. a centrally presented arrow) can be used to voluntarily direct attention toward spatial locations where an impending critical stimulus is likely to occur (or can be ignored when the cue proves uninformative). Direct comparisons of these two attentional cueing mechanisms show stronger prior entry effects with exogenous cues (Jaśkowski, 1993; Shore et al., 2001).

Averted gaze stimuli have been observed to induce strong cueing effects (Frischen et al., 2007). Yet, they fall under a unique category: while generally presented centrally in attentional orienting and TOJ paradigms (just like an arrow), they are known to elicit strong overt (Mansfield, Farroni, & Johnson, 2003; Ricciardelli, Bricolo, Aglioti, & Chelazzi, 2002) and covert (Driver et al., 1999; Friesen & Kingstone, 2003) reflexive shifts which are typically expected in response to peripheral exogenous cues. For example, enhanced discrimination has been observed for stimuli that lie in the spatial location cued by an avatar’s gaze direction (Driver et al., 1999). This was observed independently of the predictive nature of the gaze stimulus: attention was automatically drawn in the direction of the avatar’s gaze when it was noninformative, or even falsely informative, of the stimulus’ location. Similarly, gaze cueing has previously been observed to induce strong prior entry effects in TOJ tasks: PSS of peripheral visual transients are modulated by the gaze direction held by a centrally presented avatar stimulus (Schneider & Bavelier, 2003). Our task differs from this previous example since the avatar’s eyes both offer directional cues at the beginning of the trial as well as provide the transients (gaze shifts) that participants must classify as occurring first or second. Nonetheless, our Matching condition results are consistent with these previous reports (Schneider & Bavelier, 2003). Attention was automatically drawn toward the hemispace cued by the avatar’s initial gaze direction. This in turn biased TOJs in favour of the eye lying within this cued spatial location. Our Mismatching condition however also revealed that averted shifts were prioritised over direct (in the context of two hemifaces), independently of where attention was initially allocated. This implies that TOJs were not purely driven by attentional constraints and that gaze direction information was factored into the decisional process.

Thus, the determinants of TOJs can also lie in latter stages of the decisional pipeline. Previous studies have shown that under conditions of sensory uncertainty caused by saccadic suppression, some participants rely on a retrospective inferential strategy to classify the temporal order of brief visual transients (Kresevic et al., 2016). When the second of two transients coincides with a saccade, which hinders its sensory processing, this subset of participants arbitrarily evaluates the second stimulus as occurring first. The fact that only a subset of participants exhibits a temporal order reversal under these specific circumstances, while saccades impair sensory processing across the whole sample, reveals that participants default to a retrospective inference strategy when dealing with unreliable sensory information. In our study, we manipulated SOAs, thus sensory uncertainty was related to task difficulty (greater uncertainty with smaller SOAs). Our Mismatching condition revealed that, independently of where attention was initially allocated (no main effect, or interaction involving *Starting Gaze Direction*), the averted shifts were prioritised over direct shifts, thus suggesting that TOJs were informed by a retrospective inferential strategy based on the type (direct or averted) of eye shift performed. This leads to the question of what specific feature of these gaze shifts was the inference based on. One possibility is that of a velocity based criterion; averted shifts involved larger angular displacements than direct shifts. All trials began with both eyes holding averted leftward or rightward gaze: averted shifts (from the initial averted to averted in the opposite direction after the shift) were larger than direct shifts (from initial averted to direct). Averted shifts were however prioritised over direct only within a specific face context (two hemifaces), which implies that gaze information was integrated with other facial feature information. This interaction suggests that the retrospective judgements were driven by a gaze direction-based criterion as the one face/two hemiface contexts impose different constraints on directional congruency between left and right eye behaviours. While directionally mismatching behaviours are rarely observed within a single face (i.e. strabismus), they frequently occur when gaze shifts occur across faces of different individuals. Also, the prioritisation of averted shifts was not accompanied by improvements in TOJ discrimination (no reduction of *SD* values), further suggesting a postdictive strategy that operates independently of early sensory processing stages.

Despite being both relevant to social communication, several studies highlight asymmetries in the processing of direct and averted stimuli. Imaging studies have shown enhanced responses in the fusiform gyrus and amygdala for direct opposed to averted gaze (George, Driver, & Dolan, 2001; Kawashima et al., 1999), and behavioural studies have revealed a prior for direct gaze in conditions of uncertainty (Mareschal, Calder, & Clifford, 2013). When compared with averted, direct gaze is also known to enhance attention and cognition, that is, the so-called *eye-contact effect* (Senju & Johnson, 2009), and using a continuous flash suppression technique, direct gaze has been found to break through suppression faster than averted gaze (Stein, Senju, Peelen, & Sterzer, 2011). Gaze contact also improves face recognition (Hood, Macrae, Cole-Davies, & Dias, 2003; Vuilleumier, George, Lister, Armony, & Driver, 2005) and gender categorisation (Macrae, Hood, Milne, Rowe, & Mason, 2002). These asymmetries are reflected in the detection of direct and averted gaze stimuli. Visual search studies have highlighted that direct stimuli are processed faster and more accurately than averted (Conty, Tijus, Hugueville, Coelho, & George, 2006; Senju & Hasegawa, 2005; Senju, Kikuchi, Hasegawa, Tojo, & Osanai, 2008; von Grünau & Anston, 1995). We showed that, despite being processed slower, averted stimuli might be prioritised over direct when specific face context conditions are met. Averted shifts could determine prior entry effects, as these can potentially signal the presence of behaviourally relevant information in the environment. Given, however, the postdictive nature of this strategy, an alternative possibility is that direct gaze stimuli stand out more than averted stimuli, leading to a longer persistence of the former in iconic memory. This longer persistence might in turn bias judgements of temporal order where direct gaze shifts are perceived as occurring more recently (i.e. after) than the averted shifts.

## Conclusion

In this study, we identified the determinants of TOJs of gaze shift behaviours. By manipulating gaze directional cueing information (that occur prior to gaze shifts), the directional congruency between left and right eye behaviours (after the gaze shifts) and the relationship between gaze shifts and face contextual information, we isolated two mechanisms that influence gaze shift TOJs. The first involved a reflexive attentional shift induced by the avatar’s fixation direction prior to the onset of the left and right eye gaze shifts. TOJs were biased in favour of the eye lying in the hemispace (left or right) cued by the avatar’s initial gaze direction (leftward or rightward). The second involved a retrospective evaluation of temporal order where priority was assigned to a gaze shift based on its directional content and independently of where attention was initially allocated.
